# Harnessing apoptotic cells to enhance efficiency of macrophage‐based cell therapy

**DOI:** 10.1002/ctm2.70008

**Published:** 2024-10-06

**Authors:** Imke Liebold, Lidia Bosurgi

**Affiliations:** ^1^ I. Department of Medicine University Medical Center Hamburg‐Eppendorf Hamburg Germany; ^2^ Protozoa Immunology Bernhard Nocht Institute for Tropical Medicine Hamburg Germany

1

Phagocytosis of apoptotic cells by macrophages, also known as efferocytosis, is a core function of macrophages in every tissue of our body. Here the prompt elimination of dying cells ensures the reestablishment of homeostasis in physiological and pathological conditions.

By leading to the accumulation of apoptotic cells, impaired efferocytosis is indeed a key contributor to many diseases, from autoimmune conditions such as systemic lupus erythematosus to cancer. Restoring phagocytosis by inhibiting “do‐not‐eat me” signals or by blocking the programmed cell death protein 1‐programmed death‐ligand 1 (PD1‐PD‐L1) axis, increases macrophage phagocytosis of tumour cells, thereby enhancing survival in mouse models of cancer in a macrophage‐dependent manner.^1^, ^2^

Besides its involvement in the direct clean‐up of dying cells, efferocytosis also directly shapes the function of phagocytic macrophages. This complicates our understanding of the impact of the efferocytic process on the damaged environment.

It is long‐established that the engulfment of apoptotic cells by macrophages leads to the induction of molecules with immunosuppressive functions, such as interleukin (IL)‐10, transforming growth factor beta 1, prostaglandins and platelet‐activating factors while reducing the secretion of proinflammatory cytokines such as tumour necrosis factor‐alpha, IL‐1β and IL‐8.^3^, ^4^ Thus, in certain disease settings, efficient efferocytosis is required to prevent chronic inflammation. In line with this, during a helminth infection, the uptake of dying cells promotes macrophage acquisition of a tissue remodelling profile and the associated parasite clearance.^5^ Additionally, metabolites released by apoptotic cells, such as spermidine and adenosine, contribute to fostering anti‐inflammatory properties in the engulfing macrophages.^6^

Consistent with these findings, apoptotic cells and their subsequent clearance by efferocytic macrophages have uncovered numerous potential therapeutic opportunities while also bringing to light several challenges.

The beneficial consequences of administering apoptotic cells have been reported in clinical practice. Infusion of apoptotic cells as a result of extracorporeal photopheresis, a procedure that induces cell death in peripheral blood mononuclear cells by ultraviolet light exposure, has been effectively used in patients with hematologic malignancies who undergo hematopoietic cell transplantation. This helps to prevent‐ acute graft‐versus‐host disease. Based on various promising data on pre‐clinical models,^7^ the induction of apoptosis in peripheral blood leukocytes and their consequent infusion is also planned to be tested in patients with rheumatoid arthritis (NCT02903212). Despite being used in clinical settings, the mechanism by which apoptotic cell transfer prevents pathological inflammation, particularly its impact on the efferocytic process, has not yet been clarified.

Importantly, cell death induced upon damage is often not restricted to a single cell type. On this basis, in Liebold et al.,^8^ we recently described how the cellular identity of the ingested apoptotic cell differentially influences macrophage behaviour. This opens the door to defining the nature of the apoptotic cell targets as a factor that determines the outcome of efferocytosis.^8^ In an environment enriched with IL‐4, such as during the infection with the helminth *Schistosoma mansoni*, the accumulation of distinct apoptotic cells occurs in the damaged liver. Their efferocytosis by hepatic monocytes/macrophages influences the profile signature of the phagocytic cells in different ways. Via an in vitro experimental setup, we have found that macrophages acquire a tissue‐remodelling profile only after the uptake of apoptotic neutrophils, but not after stimulation in vitro with other apoptotic cells, such as hepatocytes or thymocytes. Consequently, the adoptive transfer of differentially fed macrophages in *Schistosoma mansoni*‐infected mice differentially alters the disease outcome, with only apoptotic neutrophil‐fed macrophages actively promoting parasitic egg clearance (Figure 1).

**FIGURE 1:**
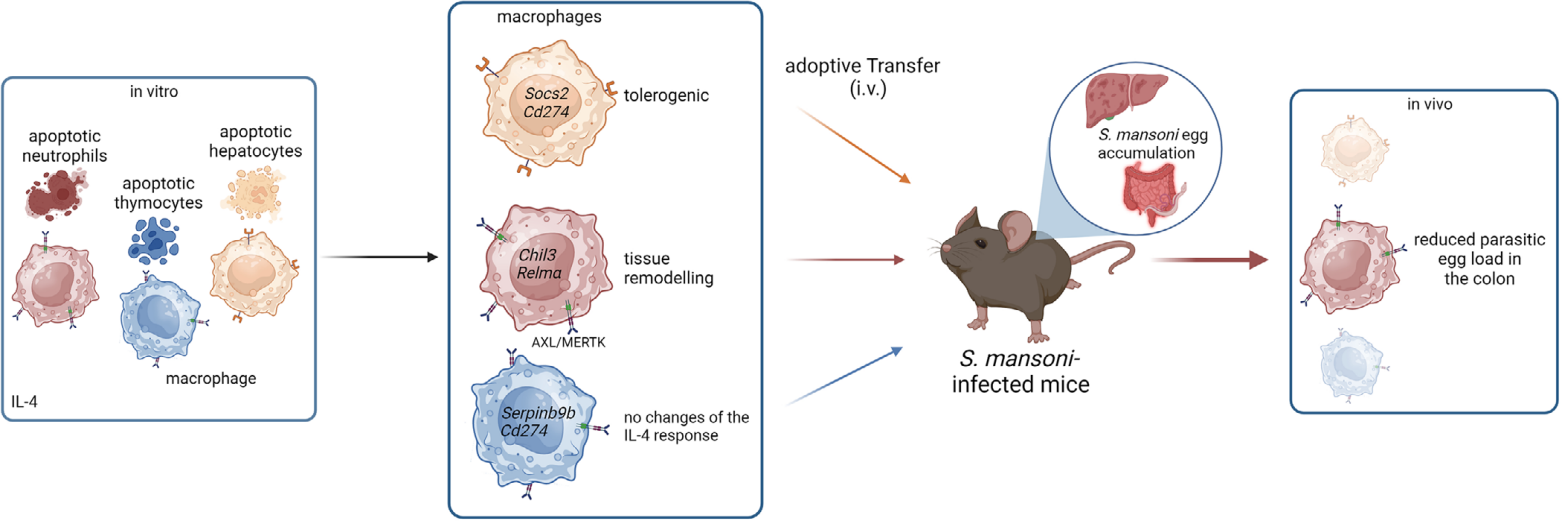
Feeding macrophages with apoptotic neutrophils increases the efficiency of macrophage‐based cell therapies in *Schistosoma mansoni* infection. Macrophages fed with apoptotic neutrophils (aN), apoptotic thymocytes (aT), or apoptotic hepatocytes (aH) and then stimulated with interleukin (IL)‐4, acquire in vitro distinct transcriptomic profiles depending on the type of apoptotic cell engulfed. Expression of genes like *Chil3* and *Rentla* is potentiated only when IL‐4‐activated macrophages engulf aN, *Socs2* and *Cd274* are induced upon uptake of aH, and *Serpinb9b* and *Cd274* upon phagocytosis of aH. Uptake of aN is mediated by the engagement of the receptor tyrosine kinases AXL and MERTK. In vivo, only the adoptive transfer of aN‐fed macrophages, but not aT or aH‐fed macrophages, reduces parasitic egg load in the colon of *S. mansoni*‐infected mice.

Based on our recent findings, the effectiveness of macrophage‐based cell therapies might be improved in a range of diseases by selective apoptotic cell feeding.

Either activation or expansion of macrophages before adoptive cell transfer has already been reported in different macrophage‐based cell therapies. Stimulation of macrophages ex vivo with recombinant human GMCSF or expansion via ixmyelocel‐T is occurring prior to autologous cell transfer in patients with ischaemic and haemorrhagic stroke (NCT01845350) and in patients with heart failure due to dilated cardiomyopathy (NCT01670981), respectively. In these scenarios, it would be worthwhile to investigate whether ex vivo feeding of monocytes/macrophages with specific types of apoptotic cells, such as apoptotic neutrophils to promote tissue remodelling, prior to macrophage adoptive transfer, could enhance clinical outcomes. This approach could pave the way for the establishment of a novel methodology in regenerative medicine.

Additionally, while our data in preclinical models still do not clarify which apoptotic cell mediators contribute to macrophage commitment toward the acquisition of certain functions, we found that there is a complementary between the identity of the apoptotic cell phagocytosed and the type of phagocytic receptor engaged. Here, engagement of the receptor tyrosine kinases AXL and MERTK preferentially leads to the uptake of apoptotic neutrophils and consequent acquisition of a tissue remodelling signature in efferocytic macrophages.^8^ This supports the idea that signalling downstream different phagocytic receptors might control the functional profile acquired by the phagocytic cell counterpart.

This concept should now be considered, especially when employing the chimeric antigen receptor for phagocytosis,^9^ which involves expressing engineered receptors in phagocytic cells to promote the engulfment and elimination of selected target cells. For instance, the engineering of macrophages with the cytosolic domain from MERTK, which has been described to trigger tumour cell cytotoxicity via targeting of immunosuppressive CCR7^+^ cells inside the tumour,^10^ might exert its effect in prolonging survival in mouse models also because of the post‐engulfment consequences of taking up apoptotic cells with specific identities.

Therefore, based on our findings, it is tempting to speculate that in the future, we could engineer macrophages to express selected phagocytic receptors based on the desired functional outcome or predict the outcome of the macrophage immune response based on the identity of the apoptotic cells located in the damaged tissue. While further studies need to be performed to address these points, our findings indicate that macrophages are pivotal in advancing cell‐based therapies due to the possibility of programming them toward specific functions by simply letting them phagocytose apoptotic cells with different identities. This versatility enhances their therapeutic potential and paves the way for promising future approaches to effectively treating various diseases for which macrophage‐based cell therapies are already ongoing.

## CONFLICT OF INTEREST STATEMENT

The authors declare no conflict of interest.

## ETHICS STATEMENT

Not applicable.

## PATIENT CONSENT STATEMENT

Not applicable.

## Data Availability

Data sharing is not applicable to this article as no datasets were generated or analysed during the current study.
